# Low Impact of Avian Pox on Captive-Bred Houbara Bustard Breeding Performance

**DOI:** 10.3389/fvets.2017.00012

**Published:** 2017-02-13

**Authors:** Guillaume Le Loc’h, Mam-Noury Amadou Souley, Stéphane Bertagnoli, Mathilde C. Paul

**Affiliations:** ^1^UMR1225 IHAP, ENVT, INP, Toulouse, France; ^2^RENECO Wildlife Preservation, Abu Dhabi, United Arab Emirates

**Keywords:** avipoxvirus, bird reintroduction, *Chlamydotis undulata*, *Chlamydotis macqueenii*, conservation breeding, display, egg production

## Abstract

Avian pox, a disease caused by avipoxviruses, is a major cause of decline of some endangered bird species. While its impact has been assessed in several species in the wild, effects of the disease in conservation breeding have never been studied. Houbara bustard species (*Chlamydotis undulata* and *Chlamydotis macqueenii*), whose populations declined in the last decades, have been captive bred for conservation purposes for more than 20 years. While mortality and morbidity induced by avipoxviruses can be controlled by appropriate management, the disease might still affect bird breeding performance and jeopardize the production objectives of conservation programs. Impacts of the disease was studied during two outbreaks in captive-bred juvenile Houbara bustards in Morocco in 2009–2010 and 2010–2011, by modeling the effect of the disease on individual breeding performance (male display and female egg production) of 2,797 birds during their first breeding season. Results showed that the impact of avian pox on the ability of birds to reproduce and on the count of displays or eggs is low and mainly non-significant. The absence of strong impact compared to what could be observed in other species in the wild may be explained by the controlled conditions provided by captivity, especially the close veterinary monitoring of each bird. Those results emphasize the importance of individual management to prevent major disease emergence and their effects in captive breeding of endangered species.

## Introduction

Avian pox is a disease caused by avipoxviruses, large enveloped double-stranded DNA viruses, known to naturally infect more than 278 avian species (1). The disease can cause significant economic losses in domestic poultry, due to decreased egg production, reduced growth, blindness, and increased mortality (2). In the wild, the infection can compromise survival and breeding success by decreasing the ability to escape predators (3), to fledge and rear chicks (4), by impairing the pairing success (5) or by increasing mortality (6). The disease has also been reported in conservation breeding of wild species such as peregrine falcons in Germany (7) or Houbara bustard species (named “Houbara”) (8, 9). However, the extent to which it may affect the reproduction efficiency of such endangered species has so far never been reported.

The African Houbara bustard (*Chlamydotis undulata*) and the Asian Houbara bustard (*Chlamydotis macqueenii*) are both vulnerable species (10). In the last decades, their populations drastically declined due to over-hunting, habitat degradation, and poaching (11). In response to this, several captive-breeding programs of Houbara have been implemented in the last decades in North Africa, the Middle East, and Central Asia (12). Due to the improvement of captive-breeding techniques and management, the size of captive flocks has progressively increased over the past 20 years from hundreds of initial founders to thousands of adult breeders, allowing to produce 20,000 juveniles each year for the largest breeding facilities (9, 12).

Due to systematic vaccination and a high biosecurity level in large-scale captive-breeding programs, incidence of avian pox is usually maintained to low level (morbidity rates ranging from 0.8 to 3.7 cases for 1,000 bird-month at risk) albeit episodic outbreaks are still recorded (9). To explore a possible effect of avian pox on breeding performance of Houbara, we analyzed data collected during two outbreaks in a captive breeding in Morocco, the Emirates Center for Wildlife Propagation (ECWP).

## Materials and Methods

One-year-old captive-bred African Houbara and Asian Houbara were studied during previously described avian pox outbreaks in Morocco, in 2009–2010 and 2010–2011 seasons, respectively (9). Asian Houbara were then temporarily bred in Morocco to benefit ECWP facilities pending the full development of conservation breeding in the United Arab Emirates. Analysis was performed in four populations: females (*n* = 689) and males (*n* = 730) of African Houbara hatched in 2009, and females (*n* = 744) and males (*n* = 634) of Asian Houbara hatched in 2010.

All birds were individually tagged (leg band), and data pertaining to their breeding and medical history were recorded. Breeding performance was assessed by counting for every study bird the numbers of days of displays (for males) or number of eggs laid (for females) during their first breeding season. This count was used as the dependent variable in the analysis. Avian pox cases were detected on a daily basis, as part of routine veterinary management and surveillance of each bird. An avian pox case was defined when nodular lesions on non-feathered areas (cutaneous pox) or yellowish lesions on the mucous membranes (diphtheritic pox) were observed. As avian pox lesions are typical and as no evidence of subclinical infection have never been described, diagnosis was based only on lesion recognitions [confirmation of avian pox were, however, performed for some cases by molecular detection of avipoxvirus DNA as previously described (13)]. Birds disease status (absence/presence) before (6–8 months of age, hereafter called “pre-season”) and during (9–14 months of age, hereafter called “season”) the breeding season was determined from the database and used as an explanatory variable (avp1 and avp2 for pre-season and season, respectively) (Table 1). Eighteen other independent variables pertaining to growth, housing, and health status were included in the analysis. Some variables were calculated separately for pre-season and season (Table 2).

**Table 1:** **Characteristics of study populations**.

Species	Cohort	Sex	Selected birds	Recruitment (%)^a^	Displays/eggs^b^	Avian pox prevalence (%)^c^
African Houbara	2009	Male	730	79.6	11.7	9.7
African Houbara	2009	Female	689	13.9	3.9	7.1
Asian Houbara	2010	Male	634	94.5	8.3	3.8
Asian Houbara	2010	Female	744	32.5	6.9	7.3

*^a^Proportion of birds having displayed or laid eggs*.

*^b^Mean number of displays/egg for recruited birds*.

*^c^Proportion of birds presenting clinical signs compatible with avian pox between 6 and 14 months of age*.

**Table 2:** **Variables used in the analysis**.

Category	Variable	Description
Housing	brank	Rank of birth
Housing	site	Site mostly occupied (enjil, missour, enjil&missour)^a^
Housing	cage	Type of cage mostly occupied (battery, cage, battery&cage)
Housing	ting1	Proportion of time spent in group during the pre-season: 100% of the time in group or less than 100% of the time in group
Housing	ting2^b^	Proportion of time spent in group during the season: 0% of the time in group or more than 0% of the time in group
Housing	sofg1	Mean size of group during the pre-season
Housing	mprop1	Proportion of males in the group during the pre-season
Housing	mvt1	Moved during the pre-season (yes/no)
Housing	mvt2	Moved during the season (yes/no)
Housing	surv^c^	Being part of surveillance program (yes/no)
Health	sick1	Sick (no avian pox) during the pre-season (yes/no)
Health	sick2	Sick (no avian pox) during the season (yes/no)
Health	avp1	Sick (avian pox) during the pre-season (yes/no)
Health	avp2	Sick (avian pox) during the season (yes/no)
Growth	w1d	Log_10_ of weight at 1 day of age
Growth	w12d	Log_10_ of weight at 12 days of age
Growth	w8m	Log_10_ of weight at 8 months of age
Growth	wg1d12d	Log_10_ of gain weight between 1 and 12 days of age
Growth	wg12d8m	Log_10_ of gain weight between 12 days and 8 months of age
Growth	wg1d8m	Log_10_ of gain weight between 1 day and 8 months of age

*^a^The two sites differ in term of altitude and thus climatic conditions*.

*^b^For the African Houbara females, this variable was coded as 100% of the time in group or less than 100% of the time in group*.

*^c^Birds being part of surveillance program were more often caught and handled*.

Analyses were performed using a generalized linear model accounting for excess of 0: a hurdle model. This model is a two-component mixture model, including a binary component that generates 0s and 1s and a second component which generates non-zero values. A two-stage process generates the zero and non-zero data. It is assumed that all the zero-valued data are generated through a single process (condition is absent, thus 0 is observed) (14, 15). A binomial error distribution and a logit link function for the zeroth part of the model as well as a negative binomial error distribution for the count part were used. Model selection was conducted using Akaike information criterion by stepwise regression.

Packages such as pscl (16), MASS (17), and lmtest (18) in R software (19) were used.

## Results and Discussion

**Figure 1:**
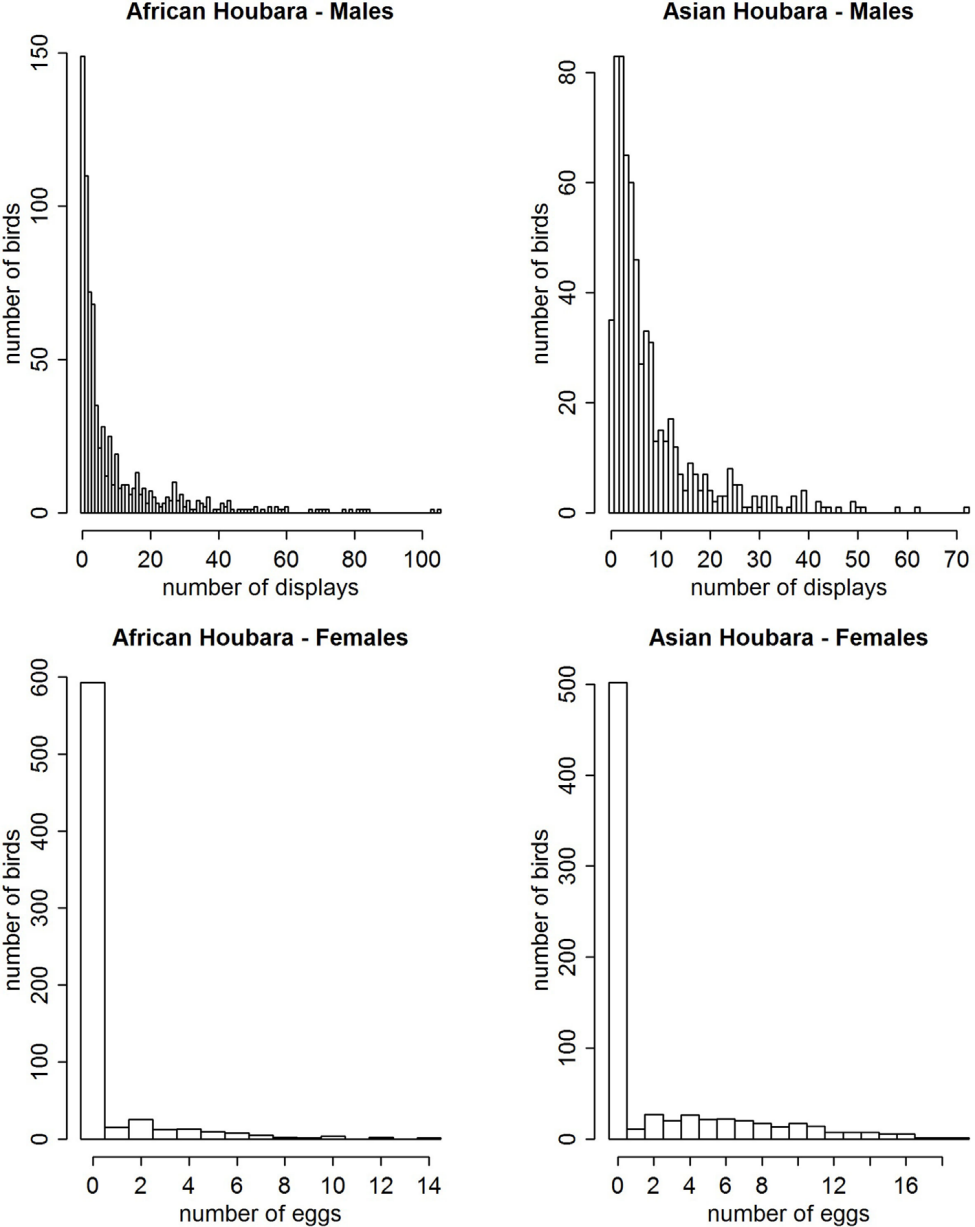
**Distribution of displays and laid eggs for the four study populations of Houbara bustard in Morocco from 2009 to 2011, during their first breeding season**.

Descriptive analysis of dependent variables showed a two-part distribution of the variables: presence or absence of displays or eggs (hereafter “recruitment”), and count of displays or eggs (hereafter “production”). Excess of 0 (recruitment ranging from 13.9 to 94.5%, Table 1) and overdispersion of the data (Figure 1) justified the use of a hurdle model.

All independent variables were initially introduced into the model. Model selection was run separately for each part of the model. First, variables were selected without forcing any of them. Then, the selection process was repeated by forcing avp1 and avp2. The two models obtained contained the same variables (±avp1 and avp2). As no significant difference was observed by likelihood ratio test between the two models, those including avp1 and avp2 were chosen as final models.

Final models showed that distribution of breeding performance was explained by growth, housing, and health, albeit only a few variables of these three categories were statistically significant (Table 3). Once adjusted on those variables, models showed that avian pox was associated with a decreased probability of recruitment and had a negative effect on the production of displays or eggs, except for Asian Houbara females for which the probability of laying at least one egg was higher when they had avian pox during the pre-season. Taken together, all effects of avian pox were weak and only significant for Asian Houbara females (Table 3).

**Table 3:** **Coefficients of variables selected in the final models for Houbara bustard breeding performance in Morocco from 2009 to 2011**.

Variable	African Houbara males		African Houbara females		Asian Houbara males		Asian Houbara females	
	Recruitment	Production	Recruitment	Production	Recruitment	Production	Recruitment	Production
brank	−0.03* (0.01)	−0.03* (0.01)			−0.02 (0.01)	−0.02* (0.01)	0.01* (0.01)	
site (missour)	−4.23* (1.20)	−0.16 (0.25)	0.04 (0.34)		na	na	na	na
site (enjil&missour)	−3.95* (1.15)	−0.56* (0.26)	−2.34* (0.52)					
cage (cage)	−2.81* (1.26)		−1.00 (0.55)		na	na	−1.66* (0.40)	−0.43* (0.14)
cage (battery&cage)	na	na	na	na	na	na	0.07 (0.46)	−0.17 (0.15)
ting1 (100%)		1.12* (0.28)		0.85* (0.31)			0.55* (0.20)	
ting2 (0%)	−3.84* (1.07)		Na	Na		−0.30 (0.17)		−0.24 (0.16)
ting2 (100%)	na	na	−2.11* (0.34)	−0.64* (0.23)	na	na	na	na
sofg1	−0.51* (0.15)	−0.35* (0.08)						
mprop1	−1.49* (0.62)		−1.15 (0.68)					
mvt1 (yes)								0.27 (0.16)
mvt2 (yes)		−1.04* (0.29)			−1.99 (1.11)			
surv (yes)	1.53 (1.01)			1.26* (0.50)				
sick1 (yes)		0.45 (0.31)	−1.11 (0.77)					
sick2 (yes)						0.40 (0.26)		−0.29 (0.17)
avp1 (yes)	−0.29 (0.49)	−0.01 (0.23)	−0.15 (0.44)	−0.17 (0.29)	−0.05 (1.08)	−0.10 (0.28)	1.31* (0.36)	−0.39* (0.20)
avp2 (yes)	−0.44 (068)	−0.60 (0.41)	−0.12 (0.95)	−1.90 (1.04)	−1.58 (1.25)	−0.92 (0.62)	−1.68 (0.86)	−0.23 (0.52)
w1d			−5.94 (3.18)	−38.43 (19.87)	−126.90 (88.05)		10.07* (2.78)	
w12d		−2.09 (1.39)	16.11* (3.69)	41.55* (20.47)	827.52* (319.78)	−2.21* (1.04)	−2.68 (1.74)	
w8m		6.29* (1.36)			−703.03* (281.03)	2.49* (1.11)		
wg1d12d		2.36* (0.94)		−24.20 (13.07)	−85.98 (61.24)	1.47* (0.69)		
wg12d8m			10.45* (2.73)	3.16 (1.82)	653.03* (259.89)			
wg1d8m	4.59* (1.74)						10.70* (1.94)	

*Coefficients are given only for variables included in the final models. Asterisks show significant coefficient (*p* < 0.05). Significant coefficients (*p* < 0.05) are highlighted in gray for avian pox variables. SEs are given in brackets. “na” indicates that the variable modality does not exist for the population*.

While the effects of avian pox have already been studied in wild birds breeding performance (4, 5), they had never been studied in captive birds, especially in conservation breeding programs. Indeed, impact of avian pox on morbidity and mortality rates has so far only been described in conservation breeding programs (7, 8) as well as in poultry production (2, 20). The present study offered a unique opportunity to assess the impact of the disease in this context. Indeed, captive breeding of thousands of individually monitored Houbara allows for the collection of large data sets enabling the measure of disease impacts at individual and flock levels.

Analyses performed during two outbreaks of avian pox showed weak effects of the disease on breeding performance, of which most of them were statistically non-significant. It has been shown in wild species that the impact of infectious disease varies with the resource availability (21). Moreover, some captive conditions such as laboratory environment can suppress the effect of infections on reproductive performance by providing controlled ambiance and resources and by limiting social interactions that can mediate the effect of pathogens (22).

In Houbara captive-breeding programs, birds are housed in individual cages or by small groups, and food and water are distributed *ad libitum*. By providing an easy access to resources without competition, one certainly helps birds to counteract disease effects.

However, this may not be sufficient to explain the absence of disease effect. The medical management of birds can be another key factor explaining the absence of observed effect of avian pox. Due to a daily control of the whole captive flock, every sick bird is detected at a very early stage and individual medical care is provided, which stops the development of debilitating lesions.

Our study showed significant effect of avian pox on Asian Houbara females for which the probability of laying at least one egg was higher, but the number of eggs laid lower when they had avian pox during the pre-season. In Houbara, it has been suggested that repeated handling and environmental enrichment can improve tameness and thus breeding performance by decreasing the stress of captivity (23). This is especially true for females that are frequently handled for artificial insemination. We can hypothesize that avian pox, when occurring before the breeding season, has an indirect positive effect due to an increased handling of birds for medical cares and thus an increased tameness. Nevertheless, this does not prevent a negative impact of the disease on the number of eggs laid as observed.

While a previous study has shown that individual management in large captive flock is not enough to fully control disease at a flock level (9), the present study has showed that individual management and cares, applied in conservation breeding projects, could allow for controlling some impacts of diseases. Further studies are needed to generalize this conclusion by assessing the impact of avian pox and other infectious diseases on a wider panel of physiological parameters such as growth or immunological status.

## Ethics Statement

This study has been performed using data collected during the daily veterinary management of captive breed of Houbara bustards. Those birds are captive bred for conservation purpose.

## Author Contributions

GL and MP designed the study. GL and M-NS performed and interpreted the analyses. GL, SB, and MP drafted the manuscript.

## Conflict of Interest Statement

The authors declare that the research was conducted in the absence of any commercial or financial relationships that could be construed as a potential conflict of interest.
